# Can Clinical Scores Reduce CT Use in Renal Colic? A Head-to-Head Comparison

**DOI:** 10.3390/tomography11100113

**Published:** 2025-10-09

**Authors:** Ramazan Kıyak, Meliha Fındık, Bahadır Çağlar, Süha Serin, Gökhan Taşkın, Ahmet Buğra Önler

**Affiliations:** Department of Emergency Medicine, Faculty of Medicine, Balıkesir University, Balıkesir 10145, Türkiyeg.taskin1983@hotmail.com (G.T.);

**Keywords:** CHOKAI, kidney, ureteral stone, modified STONE

## Abstract

Objective: Non-contrast computed tomography (CT) remains the gold standard for diagnosing ureteral stones, with excellent sensitivity and specificity. However, reliance on CT alone raises concerns regarding cumulative radiation exposure, particularly in recurrent stone formers. Clinical scoring systems such as CHOKAI, STONE, and modified STONE have been developed to provide practical bedside tools for diagnostic decision-making. This study prospectively compared these three clinical scores for their ability to predict urinary-stone disease in the emergency department. Study Design: Prospective study. Methods and Duration of the Study: Between 6 August 2024 and 15 February 2025, 130 consecutively enrolled adults with flank pain underwent bedside scoring and reference-standard non-contrast CT. Associations were analysed with Chi-Square Tests and multivariable logistic regression. Model calibration was assessed with the Hosmer–Lemeshow test; overall accuracy was calculated. Results: When the variables used in different stone scoring formulas were compared according to the computer tomography results, there was a statistically significant difference (*p* < 0.01) between patients with and without a history of stone and hydronephrosis. Patients with nausea, history of stone, and hydronephrosis were 11, 4.2, and 5 times more highly to have a stone on computer tomography than those without, respectively. Conclusions: In this Turkish cohort, CHOKAI and modified STONE demonstrated superior predictive performance compared to the original STONE score. These findings suggest that clinical scoring systems, when incorporating predictors such as nausea, prior stone history, and hydronephrosis, may serve as practical alternatives to CT-first diagnostic approaches. Multicenter validation studies are required before routine clinical adoption.

## 1. Introduction

Renal colic is among the most intense and rapidly progressive types of visceral pain encountered in clinical practice, most commonly caused by acute ureteral obstruction due to calculi, and it accounts for a considerable proportion of emergency department presentations worldwide [1]. Urolithiasis is the most common urological emergency, representing a major cause of non-traumatic flank pain presentations in emergency departments worldwide. Its incidence has been rising over recent decades, largely attributed to lifestyle modifications, dietary habits, and associated metabolic changes [2]. In Türkiye, the prevalence of urinary stone disease is estimated at approximately 14%, positioning the country among those with a relatively high burden of urolithiasis. This elevated prevalence is likely influenced by regional dietary patterns, climatic conditions, and genetic predisposition [3].

Kidney stones can be classified based on their chemical composition, with calcium oxalate stones being the most common, followed by uric acid, struvite, and cystine stones. While the underlying composition has therapeutic and prognostic implications, the acute clinical presentation is often similar across stone types. An additional advantage of CT is its ability to detect stones regardless of their chemical composition, including uric acid stones, which are radiolucent and not visible on plain X-ray images. Several imaging modalities are available for evaluating suspected urolithiasis. Ultrasound is frequently used as a radiation-free and cost-effective tool; plain radiography may detect radiopaque stones, and magnetic resonance urography provides additional functional information without radiation exposure. Nevertheless, non-contrast CT, including low-dose protocols, remains the most accurate and widely used imaging technique in emergency settings.

A range of imaging modalities, including ultrasound, plain radiography, and non-contrast computed tomography (CT), are routinely employed in the evaluation of patients with renal colic. Among these, CT remains the most accurate technique for detecting urinary calculi, whereas ultrasound provides a radiation-free, cost-effective, and readily available initial assessment tool [4]. Among these modalities, non-contrast computed tomography has emerged as the gold standard imaging technique in emergency settings, offering excellent sensitivity (approximately 95–98%) and specificity (>96%) for the detection of urinary calculi [5]. However, the widespread use of CT raises significant concerns regarding cumulative radiation exposure, particularly among recurrent stone formers. In the United States alone, emergency departments perform an estimated 1.6–2.1 million CT scans annually for suspected ureterolithiasis, substantially contributing to the cumulative radiation dose in this patient population [6].

Since urolithiasis is a chronic and recurrent condition with recurrence rates reported to be as high as 50% within 5–10 years, patients often require repeated imaging, which contributes to cumulative radiation exposure and increases the risk of potential long-term adverse health effects [7]. To enhance diagnostic efficiency and reduce unnecessary imaging in patients with suspected urolithiasis, several clinical scoring systems, such as CHOKAI, STONE, and the modified STONE score have been developed and applied in emergency department settings. Among these, the CHOKAI score has demonstrated superior diagnostic accuracy, while the modified STONE score has shown strong predictive value across diverse patient populations [8]. To optimize diagnostic efficiency and minimize unnecessary imaging in patients with suspected urolithiasis, several clinical scoring systems, such as CHOKAI, STONE, and the modified STONE score have been implemented in emergency department practice. These decision-support tools not only help reduce cumulative radiation exposure from CT scans but also shorten emergency department length of stay without compromising diagnostic accuracy [9].

While previous studies have evaluated the CHOKAI and modified STONE scores individually or in pairs, to our knowledge, no prospective study has performed a direct, head-to-head comparison of the CHOKAI, original STONE, and modified STONE scores within the same patient cohort. Applying these three clinical prediction tools simultaneously under standardized clinical conditions enables a robust assessment of their relative diagnostic performance. Considering that the accuracy of clinical scores may vary according to demographic and geographic factors, our study also addresses the current gap in the literature by validating these tools in a Turkish emergency department population. This comparison is intended to guide clinicians in selecting the most effective tool for minimizing unnecessary CT utilization in patients presenting with suspected renal colic.

The CHOKAI scoring system integrates multiple clinical parameters, including nausea/vomiting, hydronephrosis, hematuria, prior history of urinary stones, gender, age, and pain duration to stratify the likelihood of ureteral stones. A total score of 0–5 reflects low risk, whereas a score of 6–13 suggests a high probability of stone presence [8]. The STONE score is a widely utilized clinical prediction model that incorporates five key variables: gender, duration of pain, ethnicity, presence of nausea or vomiting, and hematuria to estimate the probability of ureteral stone disease. Based on the cumulative score, patients are stratified into low (0–5), moderate (6–9), and high (10–13) risk categories [10]. The modified STONE scoring system enhances diagnostic accuracy by including five parameters gender, pain duration, hematuria, history of urinary stones, and C-reactive protein (CRP) levels and stratifies patients into low (0–4), moderate (5–9), and high (10–16) risk categories, demonstrating improved performance with an AUC (Area Under the Curve) of 0.94 in the original cohort and consistent external validation across various populations, while aligning with contemporary biomarker-based triage strategies [11,12]. Although the components of the CHOKAI, STONE, and modified STONE scores differ in certain aspects, their clinical parameters are summarized consecutively in the text to allow straightforward comparison without the need for an additional table.

In current literature, most studies evaluating clinical prediction tools for urinary stones have primarily focused on comparing two scoring systems or establishing optimal cut-off values for individual models [13]. What distinguishes the present study is its comprehensive, head-to-head comparison of three validated scoring systems, CHOKAI, STONE, and modified STONE, within a single cohort, which is relatively rare and addresses a notable gap in evidence [14]. The present study aimed to assess and compare the diagnostic performance of the CHOKAI, STONE, and modified STONE scoring systems in a real-world emergency department population with suspected renal colic; such side-by-side validation aligns with recent Frontiers research highlighting the clinical utility of integrated decision support tools in ED settings [15]. By correlating each score with non-contrast CT findings, the study sought to elucidate the predictive value of individual clinical variables incorporated into these models, reflecting a diagnostic pathway that integrates clinical prediction tools with imaging outcomes an approach supported by recent Frontiers research on clinical decision support systems in imaging [16].

## 2. Methodology

This prospective, single-center study was conducted in the Emergency Department of Balıkesir University Faculty of Medicine, a tertiary care academic hospital. The study was conducted between 6 August 2024, and 15 February 2025. This was a prospective, observational design, and all patients were evaluated under the same institutional protocol. Consecutive adult patients presenting between 6 August 2024, and 15 February 2025, with clinical suspicion of urinary stone disease were systematically evaluated and enrolled.

Inclusion criteria were age ≥ 18 years, flank pain consistent with suspected renal colic, and indication for non-contrast CT. Exclusion criteria included: known alternative diagnosis (e.g., appendicitis), pregnancy, recent trauma, or refusal to participate. No eligible patient was excluded selectively, minimizing selection bias. In addition to demographic and clinical data, potential risk factors predisposing to urolithiasis (such as dietary habits, medication use, recurrent urinary tract infections, or obstructive uropathy) were recorded during history-taking. However, these variables were not analyzed as independent predictors, as the study protocol primarily focused on clinical parameters included in the CHOKAI, STONE, and modified STONE scores.

The study protocol was reviewed and approved by the Balıkesir University Non-Interventional Clinical Research Ethics Committee (Approval No: 2024/120), in accordance with the principles of the Declaration of Helsinki. In addition to routine clinical evaluation, including physical examination and standard laboratory tests (blood and urine analyses), non-contrast CT was performed for all participants to confirm the presence or absence of urinary stone disease, serving as the diagnostic gold standard. CT scans were acquired with a non-contrast low-dose protocol (120 kVp, 100–150 mAs) using a slice thickness of 5 mm.

All scoring using CHOKAI, STONE, and modified STONE was performed before CT interpretation. CT scans were read by radiologists blinded to the scoring results, and emergency physicians calculating the scores were also unaware of CT findings at the time of scoring.

Statistical analysis was performed using IBM SPSS Statistics version 25.0. Descriptive statistics were used for categorical variables. Although no formal power analysis was performed, the sample size of 130 patients was comparable to or exceeded that of similar validation studies in the literature, and it was sufficient to detect statistically significant differences in AUC values between the scoring systems. Differences between groups were analyzed using Chi-square tests. Binary logistic regression was used to identify independent predictors of stone presence. ROC analysis was conducted for each score, and AUCs were compared using the DeLong method. Model fit was assessed using the Hosmer–Lemeshow test, and overall accuracy was calculated. A *p*-value < 0.05 was considered statistically significant.

## 3. Results

Of the applicants in our study, 86 were male (66.2%). The rate of patients under the age of 60 was 75.4%. The majority of patients reported pain lasting longer than 24 h (42.3%). Nausea was present in 116 patients, vomiting in 71 patients, and hematuria in 86 patients.

Among the patients who underwent CT, the number of those with stones measuring 5 mm or less was 42 (32.3%).

When comparing the variables used in different stone scoring formulas according to CT results, a statistically significant difference was found between patients with and without a history of stones and hydronephrosis (*p* < 0.05). No significant difference was found in other variables (gender, age, duration of pain, nausea, vomiting, hematuria, and CRP level) (Table 1).

When analyzing the parameters of the logistic regression model with the CT result (presence or absence of a stone) as the dependent variable, no statistically significant difference was found in gender, age, duration of pain, vomiting, hematuria, and CRP levels (*p* > 0.05). However, statistically significant differences were found in nausea, history of stones, and hydronephrosis variables (*p* < 0.05). According to these results, patients with nausea, a history of stones, and hydronephrosis are respectively 11, 4.2, and 5 times more highly to have a stone in CT results compared to those without (Table 2).

The goodness of fit of the model established as a result of logistic regression analysis was found to be consistent with the Hosmer and Lemeshow Test (Chi-Square = 8.813; *p* = 0.358). The overall correct classification rate of the model was found to be 80% (Table 3).

Urinary stones were found in 42.35% of patients with high CHOKI evaluation score, 40.77% of patients with moderate STONE score, and 35.38% of patients with high Modified STONE score. This result shows that Modified STONE and CHOKAI scoring values provide more sensitive results than STONE (Table 4).

## 4. Discussion

Emergency departments are among the most dynamic and resource-intensive units in healthcare systems, where diagnostic and therapeutic interventions are frequently focused on patients presenting with unstable clinical conditions or those at risk of rapid clinical deterioration if timely management is not initiated [17]. In routine clinical practice, a thorough patient history and meticulous physical examination remain the cornerstone of clinical assessment, serving as essential guides for diagnostic reasoning and risk stratification in emergency medicine [18]. Emergency physicians frequently rely on clinical scoring systems to augment diagnostic accuracy, particularly in high-pressure environments; tools with high sensitivity are especially valuable in ruling out critical conditions in patients with low pre-test probability, thus reducing unnecessary testing [19]. In emergency settings where rapid diagnosis is essential, clinical scoring systems such as CHOKAI, STONE, and the modified STONE score enable physicians to make prompt and accurate decisions, facilitating effective patient triage and reducing reliance on immediate CT imaging [20]. Clinical scoring systems not only enhance diagnostic sensitivity but also offer valuable insights into disease progression and inform management strategies, thereby underscoring their utility beyond initial triage in emergency department settings [21]. By enabling the exclusion of low-risk patients, clinical scoring systems help minimize unnecessary diagnostic testing, shorten emergency department length of stay, and streamline overall patient flow [22].

In this study, the diagnostic validity of CHOKAI, STONE, and Modified STONE scoring systems was evaluated in patients presenting to the emergency department with a suspected diagnosis of urinary stones. The rates of stone detection on CT were compared according to risk groups. Additionally, the variables used in these scoring systems were assessed based on CT results, and variables influencing the probability of urinary stones were examined through logistic regression analysis.

The CHOKAI scoring system incorporates seven clinical parameters: nausea or vomiting, hydronephrosis, hematuria, history of urinary stones, gender, age, and pain duration. Based on the cumulative score, patients are categorized as low risk (0–5 points) or high risk (6–13 points) for ureteral stones [23]. In the present study, urinary stones were identified in 55 of 78 patients (70.5%) categorized as high risk according to the CHOKAI score, marking the highest detection rate among all three scoring systems evaluated. This result aligns with findings from a retrospective analysis of 157 Turkish patients, which likewise reported that the CHOKAI system outperformed both the STONE and modified STONE scores in predicting ureteral stones [3]. In another prospective study comparing the diagnostic performance of the STONE and CHOKAI scores, the CHOKAI system demonstrated superior accuracy, particularly in correctly identifying patients with ureteral stones [14].

The STONE scoring system comprises five clinical parameters: gender, pain duration, ethnicity, presence of nausea or vomiting, and hematuria. Based on the total score, patients are stratified into low risk (0–5 points), moderate risk (6–9 points), and high risk (10–13 points) categories for ureteral stone presence [10]. In our study, 55 out of 93 patients in the intermediate and high-risk groups were diagnosed with urinary stones (59.1%). Hernandez et al. recommended that the STONE scoring system be primarily utilized for the evaluation of patients in the low- and intermediate-risk categories, as its diagnostic performance is less reliable in high-risk presentations [24]. In our study, 73 out of 125 patients in the low and intermediate-risk groups were diagnosed with stones (58.4%), showing that the detection rates of urinary stones were similar in both medium-high and medium-low risk groups. Cochon et al. reported that the diagnostic utility of the STONE score was limited in high-risk patients, and that incorporating hydronephrosis as an additional parameter significantly improved its predictive accuracy. Our findings support this observation, as hydronephrosis emerged as one of the strongest independent predictors of stone presence in our cohort [25].

The modified STONE scoring system incorporates five clinical parameters gender, duration of pain, hematuria, history of urinary stones, and C-reactive protein (CRP) levels to improve diagnostic precision. Patients are stratified into low (0–4 points), moderate (5–9 points), and high risk (10–16 points) categories based on the total score [11]. In our cohort, 46 of 64 patients (71.9%) classified as high risk according to the modified STONE score were diagnosed with urinary stones. Additionally, 68 of 111 patients (61.3%) in the moderate-risk group also had confirmed stone disease, highlighting the scoring system’s utility in identifying patients with a significant probability of urolithiasis.

When evaluating the risk stratification performance of these three systems, our data showed that the CHOKAI score had the highest predictive yield (42.35%) in its high-risk category, while the STONE score presented a similar yield (40.77%) in its medium-risk group indicating poor risk differentiation. Moreover, the modified STONE system, despite its newer design, had the lowest predictive value (35.38%) in the high-risk group. These findings highlight CHOKAI’s relative advantage, possibly due to its inclusion of critical variables like hydronephrosis and stone history. Yet, none of the scores are conclusive alone, and high clinical suspicion should still prompt imaging. This reinforces the idea that scoring systems are supportive rather than definitive tools in emergency settings. It should also be noted that only 5 patients in our study were classified as high risk according to the STONE score, which considerably limits the precision of comparisons in this subgroup

The majority of patients included in our study were diagnosed with urinary stones. This can be attributed to the clinical experience of the physicians, who appropriately selected patients for imaging.

When the variables from three different scoring systems were compared with CT results, statistically significant differences were found for a history of urinary stones and the presence of hydronephrosis (*p* < 0.01). No significant differences were observed for gender, age, duration of pain, nausea, vomiting, hematuria, or CRP levels. According to logistic regression analysis, nausea, history of urinary stones, and hydronephrosis were statistically significant predictors of stone detection on CT (*p* < 0.05). These findings suggest that patients with nausea were 11 times more likely, those with a history of urinary stones 4.2 times more likely, and those with hydronephrosis 5 times more likely to have urinary stones on CT.

In a retrospective study conducted in the Turkish population, male gender, hematuria, a family history of urinary stones, nausea, and vomiting were identified as significant predictors of urinary stone presence. These findings are consistent with our results, reinforcing the diagnostic relevance of these clinical parameters in emergency settings [26]. Previous studies in the Turkish population have also compared these scoring systems. Eraybar and Yuksel prospectively evaluated the diagnostic effectiveness of CHOKAI and STONE, reporting superior accuracy for CHOKAI [27]. Similarly, Bahadirli et al. demonstrated the utility of the modified STONE score in patients with flank pain [28]. In addition, Ok and Durmuş externally validated the CHOKAI and STONE scores in an Eastern Turkish cohort, further confirming their applicability [29]. These findings are consistent with our results, which support the greater diagnostic value of CHOKAI, and modified STONE compared to the original STONE score.

In our study, the Modified STONE and CHOKAI scoring systems were found to be more advantageous for clinical use in diagnosing urinary stones in the emergency department. Comparative analysis demonstrated that the CHOKAI and modified STONE scores had higher diagnostic accuracy than the original STONE score. The area under the curve (AUC) for CHOKAI was significantly greater than for the original STONE score (*p* < 0.01), supporting its superior performance. However, since no clinical scoring system achieves perfect diagnostic accuracy, the incorporation of additional clinical indicators such as nausea, prior history of urinary stones, and hydronephrosis into existing models has been shown to significantly improve risk stratification and diagnostic precision [30]. Therefore, modifying existing systems or developing new scoring systems could be beneficial. Although this is a single-center observational study and may be subject to certain limitations such as lack of randomization or external validation, the prospective design, consecutive patient enrollment, use of gold-standard CT, and blinded assessments collectively reduced the risk of bias and improved internal validity. Therefore, our results should be interpreted cautiously and confirmed through future multicenter studies with larger and more diverse patient populations.

It is important to note that ultrasonography is not universally accessible in all healthcare settings. However, in institutions equipped with radiologists or trained emergency medicine specialists, the evaluation of hydronephrosis via point-of-care ultrasound can significantly enhance the diagnostic performance of clinical scoring systems by providing real-time confirmation of urinary tract obstruction [31].

### Study Limitations

This study has several limitations. First, although ultrasound is widely used in many emergency departments because it is radiation-free and cost-effective, its diagnostic accuracy is limited and it requires real-time evaluation by an experienced radiologist or a physician trained in point-of-care ultrasound. In many hospitals, access to ultrasound is not consistently available at all times, particularly outside regular working hours, and interpretation often depends on the immediate availability of radiology staff. In contrast, non-contrast CT scanners are increasingly available in tertiary and secondary centers, and CT images can be interpreted on-site or remotely, which enhances reliability and continuity of diagnostic services. For these reasons, despite the disadvantages of radiation exposure and cost, CT remains the gold standard for the diagnosis of kidney stones due to its superior sensitivity, specificity, and broader accessibility. Second, this study was conducted in a single center in Turkey. The prevalence and risk factors for urolithiasis may differ across geographic regions, which may limit the generalizability of our findings. Therefore, multicenter and international studies are warranted to validate our results in more diverse populations.

## 5. Conclusions

All scoring systems studied were found to be effective in diagnosing urinary stones in the Turkish population. However, additional findings such as a history of urinary stones, nausea, and hydronephrosis in the existing scoring systems increase diagnostic accuracy. To reduce radiation risks associated with repeated CT imaging, scoring systems should be revised to include such symptoms. This approach will help avoid time and resource waste and improve patient management. In this single-center prospective study, both CHOKAI and modified STONE scores demonstrated higher diagnostic performance compared to the original STONE score. Furthermore, nausea, hydronephrosis, and a history of urinary stones were identified as independent predictors of stone presence on CT. These findings suggest that improved scoring systems may help prioritize patients and potentially reduce unnecessary CT use. However, multicenter validation studies are required before widespread clinical implementation.

## Figures and Tables

**Table 1:** Comparison of variables used in different stone scoring formulas based on CT results.

Variables		Results of CT (Stone)		X^2^	*p*
		Yes	No		
Gender	Male	53	33	1.612	0.261
	Female	22	22		
Age	>50	39	27	0.107	0.948
	50–60	18	14		
	>60	18	14		
Pain Duration (Hours)	>6	14	16	1.992	0.369
	6–24	28	17		
	>24	33	22		
Nausea	Yes	64	52	2.802	0.080
	No	11	3		
Vomiting	Yes	41	30	0.000	0.565
	No	34	25		
History of Stones	Yes	47	19	10.039	0.001 *
	No	28	36		
Hematuria	Yes	53	33	1.612	0.140
	No	22	22		
Hydronephrosis	Yes	52	8	38.325	0.001 *
	No	23	47		
CRP (mg/dL)	<0.05	29	23	0.131	0.427
	≥0.05	46	32		

* *p* < 0.05.

**Table 2:** Logistic Regression Analysis of the Probability of Having a Stone Based on CT Results Using Different Stone Scoring Variables in Patients Suspected of Urinary Stone.

Variable	Β	S.E.	Wald	*p*	Odds Ratio
Gender	−0.401	0.518	0.599	0.439	0.670
Age					
>50			3.828	0.147	
50–60	−1.256	0.678	3.436	0.064	0.285
>60	−0.481	0.738	0.424	0.515	0.618
Pain Duration					
>6			2.770	0.250	
6–24	0.816	0.719	1.287	0.257	2.261
>24	−0.281	0.619	0.206	0.650	0.755
Used Variables in Formulas					
Nausea	2.416	0.890	7.372	0.007 *	11.205
Vomiting	−0.048	0.527	0.008	0.923	1.055
History of Stones	−1.427	0.545	6.857	0.009 *	4.240
Hematuria	−0.054	0.551	0.009	0.923	1.055
Hydronephrosis	−2.965	0.554	28.615	0.001 *	5.052
CRP (mg/dL)	0.401	0.526	0.583	0.445	1.494

* *p* < 0.05.

**Table 3:** Percentage Classification of Stone Detection in Patients Suspected of Urinary Stone.

Observed	Predicted—Stone Present	Predicted—Stone Absent	Percentage Correct
Stone Present	62	13	82.7
Stone Absent	13	42	76.4
Overall			80.0

**Table 4:** Comparison of CHOKAI, STONE, and Modified STONE Score Values with CT Results.

Scoring	Levels	Stone Present		Stone Absent		Total	
		%	f	%	f	%	f
CHOKAI Risk	Low	15.38	20	24.62	32	40.00	52
	High	42.35	55	17.69	23	60.00	78
STONE Risk	Low	15.38	20	13.08	17	28.46	37
	Medium	40.77	53	26.92	35	67.69	88
	High	1.54	2	2.31	3	3.85	5
MODIFIED STONE Risk	Low	5.38	7	9.23	12	14.62	19
	Medium	16.92	22	19.23	25	36.15	47
	High	35.38	46	13.85	18	49.23	64

## Data Availability

The data presented in this study are available on request from the corresponding author. The data are not publicly available due to privacy and ethical restrictions.
